# Trustworthy Intrusion Detection in E-Healthcare Systems

**DOI:** 10.3389/fpubh.2021.788347

**Published:** 2021-12-03

**Authors:** Faiza Akram, Dongsheng Liu, Peibiao Zhao, Natalia Kryvinska, Sidra Abbas, Muhammad Rizwan

**Affiliations:** ^1^Department of Mathematics, School of Science, Nanjing University of Science and Technology, Nanjing, China; ^2^Department of Information Systems, Faculty of Management, Comenius University in Bratislava, Bratislava, Slovakia; ^3^Department of Computer Science, COMSATS University, Islamabad, Pakistan; ^4^Department of Computer Science, Kinnaird College for Women, Lahore, Pakistan

**Keywords:** network security, privacy, ANFIS, intrusion detection, IoT based networks

## Abstract

In Internet of Things (IoT)-based network systems (IoT-net), intrusion detection systems (IDS) play a significant role to maintain patient health records (PHR) in e-healthcare. IoT-net is a massive technology with security threats on the network layer, as it is considered the most common source for communication and data storage platforms. The security of data servers in all sectors (mainly healthcare) has become one of the most crucial challenges for researchers. This paper proposes an approach for effective intrusion detection in the e-healthcare environment to maintain PHR in a safe IoT-net using an adaptive neuro-fuzzy inference system (ANFIS). In the proposed security model, the experiments present a security tool that helps to detect malicious network traffic. The practical implementation of the ANFIS model on the MATLAB framework with testing and training results compares the accuracy rate from the previous research in security.

## Introduction

Internet of Things (IoT)-based network systems (IoT-net) are considered as emerging advancements in the field of technology, where cloud network-based servers provide communication, storage, and problem-solving facilities, but these sorts of systems also contain security threats and issues as well (1–4). The network user can access its facility by using an internet source (5). The multiple hardware and software-based environments provide data and information to its end users. Some of the most prominent organizations like Apple, HP, Amazon, IBM, Oracle, Intel, etc., use cloud computing (CC) techniques. CC is based on three-layer models: Infrastructure as a Service (IaaS), Platform as a Service (PaaS), and Software as a Service (SaaS). Similarly, cloud networks are based on four types: private, public, hybrid, and community (6, 7).

In a cloud networking environment, problems in security and authorization are the key risks. Likewise, there are numerous risks which users and network service providers both face. Mainly, security issues arise from the data storage and networking side (8, 9). Many security enhancement-based algorithms have been proposed to make the IoT-net servers secure. Many cryptographic algorithms like RSA, AES, CRT-RSA, DES, blockchain, machine learning, and artificial intelligence-based code have been proposed to enhance security. Homomorphic encryption algorithms (10–12) help to provide better security to detect non-authorization factors. Many machine learning (13) and artificial intelligence-based security algorithms help to provide better data networking with new classification and risk assessment techniques. However, many elements remain unsolved and require more improvement and advancement in areas such as cost management, resource utilization, speed prediction, and security, most importantly.

IoT also plays a magnificent role in the healthcare sector concerning data storage, online systems, software, laboratories, pathology, clinic sessions, etc. Medical patient health records (PHR) are an emerging version of IT and smart healthcare records (5). However, here we encounter problems of data security and privacy of PHR (1, 14–18). To overcome the PHR security issue, many researchers have been working on using blockchain and many other artificial intelligence techniques, which allows clinical expertise, patients, laboratories, and the world to be connected.

One of the most effective artificial intelligence techniques is called the artificial neural inference system (ANFIS), which is the combination of an artificial neural network (ANN) and a fuzzy inference system (FIS). ANFIS is mainly used as a computational model for resolving uncertainties, reasoning, and reducing security threats from networks and cloud servers (1). It helps the systems and servers examine the risk of data and information. ANNs work on the principle of mathematical calculation. The ANFIS model is based on if-then rule statements and crisp values, and the final ANFIS surface model uses result accuracy rate. It uses most of its computational time for data classification and estimation.

### Problem Statement

In smart e-healthcare systems, networking servers provide a better intermediary platform for data storage, communication, and many other aspects. The end users (doctors, clinical experts, patients, laboratories) get the opportunity to access the PHR and access the cloud servers. It can be any authorized person. It is essential to detect and classify malicious activities and network traffic. The classification of network traffic and attack type detection helps make the system more secure and detect network intrusion.

### Motivation

In this research paper, we discuss the existing security risks in IoT-net-based PHR. We classify these issues in managing risk, end-user risk, organizational risk, privacy risk, and many more. Our main agenda is to propose a less risky cloud network for everyone. We represent the list of shortcomings in existing malicious network traffic, and this will benefit the networking service provider and security handler community to comprehend the security issue.

### Contribution

In this research, we try to implement the ANFIS model to detect network attacks on database servers. The main agenda is to detect the unauthorized access of users by using an ANFIS-based intrusion detection system. The proposed security algorithm helps to collect malicious activities or information from network traffic. Based on the if-then rule statement and ANFIS-based data classification which can help detect the intrusion attack, the system can determine if the cloud server has been hit by an intruder or not. If there is an attack, identifying the type of attack helps researchers implement precautionary measures to overcome the loss or block the malicious incoming traffic.

### Organization

The rest of the research paper is organized as follows: The section literature review discusses the literature review of the previously proposed security approaches. After that, section proposed methodology discusses the proposed work with the working of ANFIS and the proposed security algorithm along with its method. Similarly, section experimental analysis and results discusses the implementation, results, and discussion. Moreover, lastly, section conclusion discusses the conclusion and future work.

## Literature Review

According to previous research (1), healthcare-based networking servers require more security and a safe network architecture to provide confidentiality, integrity, privacy, and authentication to its patients, doctors, and management. The author proposed the ANFIS-based data classifier and security provider tool or model for making cloud servers more secure with a higher accuracy rate and smaller error rate. Detection of security attacks and malicious network traffic is considered as the most topical issue for network security (19–23). Therefore, previous research (24) presented an ANFIS-based security framework for classification of attacks and identifying their type.

In another study (25), the research mainly focuses on the interoperability of the cloud server platform and its reliability. The paper uses the hybrid approach of the squirrel search genetic algorithm with the combination of ANFIS to perform better functionality and remove uncertainties of servers by providing a higher accuracy rate. Also, in a previous work (26), an SDN anomaly detection system was proposed to detect malicious behavior and intrusion attacks using the cloud medium. The detection system identified the trusted edge for data or information sharing and communication. Based on multiple parameters, the implementation of the proposed systems showed better performance results. Here Table 1 represents the ANFIS methodology used for security purposes like in clustering, classification, accuracy, performance evaluation, etc.

**Table 1:** Recent research on ANFIS and network security issues.

**References**	**Type of risks**	**Approaches**
Srilakshmi and Muthukuru (27)	Worm-hole and malicious nodes	Hybrid reactive search and bat (HRSB) mechanism used to detect malicious nodes and ANFIS for testing and training data
Pawar and Jagadeesan (28)	Black-hole attack	Used a self-adaptive multi-verse optimizer with ANFIS to detect intrusion attacks in WSN
Maheswari and Karthika (29)	Intrusion detection in network	ANFIS clustering methodology used for selecting cluster heads
Parfenov et al. (24)	Denial of service attack	ANFIS used to improve network traffic attack detection and performance evaluation
Nandi and Kannan (30)	Packet flooding attacks	ANFIS classifier used for feature extraction and classification in MANET
Hemalatha et al. (31)	Routing attack	ANFIS used for initial feature selection and trust evaluation
Barraclough et al. (32)	Phishing attacks	ANFIS-based classification approach for higher accuracy rate

In many previous kinds of research, the communication between devices and multiple systems can also cause security threats and need efficient and enhanced network capacities during communication. Hence in a previous research (33), the author used 5G and the multiple-input multiple-output (MIMO) concept to enhance the network capacity and area coverage capacity. Similarly, in another paper (19), the author proposed the ANN technique to detect and identify intrusion attacks in android systems.

In a previous study (34), the author dealt with the security factor of the cloud to improve its performance using the AI technique. The proposed approach helped to overcome the flaw of data breaching and non-authorization access on cloud servers. They utilized different labels to restrict access to specific edge limits to ensure accuracy in decision making. It helped the systems to store a large amount of data securely on cloud servers.

In an earlier paper (35), the study presented fuzzy-based detection schemes by various machine learning techniques and data mining algorithms to cope with multiple types of malicious attacks or intrusion. This paper first categorized their contribution in two parts, intrusion and detection, and then used fuzzy techniques for classification and identification. Then they listed the shortcomings and merits of the ID detection based on fuzzy techniques and algorithms.

In another research paper (36), the author presented a detailed survey on anomaly detection schemes and fuzzy inference systems. This paper mainly focused on combining fuzzy inference systems and machine learning algorithms for the intrusion detection process. It summarized the research with the contributions and shed light on the shortcomings and benefits of FIS. Then, finally, it concluded with future findings and issues in the anomaly detection scheme.

CC is the computing solution that allows multiple end users to connect, share, communicate, and store data or information. Fault tolerance is considered the main concern in data reliability. Therefore, in a previous research (37), detailed analysis was presented to detect the nature of the error and proper implementation of the FIS technique to the response. The process of checkpoints was used to check the intensity of the error.

Table 2 represents the methodology used for cloud security based on previous research work.

**Table 2:** Recent research on network security risks.

**Types**	**Risks**	**Approaches**
Data security	Unauthorized access, data leakage, data disclosure, privacy disclosure	Hybrid approach of the DSA algorithm with reverse flow (38). Hybrid encryption algorithm for securing big data storage (39).
Platform security	Data sharing, software, hardware, application	Combination of AES, Blowfish and Twofish security algorithms for secure data sharing (40). Enhanced role-based access algorithms to secure IoT-based cloud (41).
Application security	Configuring, system accessing, management	Hybrid framework of ECC and AES for advance encryption approach (42). Publisher subscriber algorithm with ontology logic for encryption to maintain confidentiality and authenticity (43).
Infrastructure security	Cloud framework, fault injection, false model, resource handling	Proposed RDFI strategies with chaos engineering algorithm (CEA) for deployed infrastructure (44). Used CSBAuditor to monitor and detect malicious attacks in the cloud environment (45).
Physical security	Sensitive data leakage, privacy breaching, hacking, intrusion	Lightweight cryptographic algorithms for physical security analysis (46). SHA-512, ECC, and RSA hybrid cloud security system used for securing data in a cloud structure (47).

In earlier research (48), a comprehensive review discussed CC and IoT in the healthcare sector including smart systems, smart applications, smart software, smart hospitals, and smart record systems. The study presented the IoT-based smart cloud paradigm in the healthcare sector. Similarly, In an earlier work (49), an ANFIS-based healthcare system was proposed to avoid network security risks. The proposed system and already existing ANFIS-based system were compared to evaluate the performance.

Based on previous work, ANFIS is considered an advanced technique for data evaluation, classification, clustering, increased accuracy rate, and detecting various network or systems attacks or any malicious activities on cloud infrastructure to provide a trustful environment to its end users.

## Proposed Methodology

We propose a tuned version of the ANFIS model to detect intrusion attacks in cloud database servers. We mainly focus on identifying the unauthorized access of end users to systems and diagnosing the type of network attack. The proposed security algorithm will help systems to detect malicious activities or irrelevant information from network traffic. The coming subsection will help understand the ANFIS model's working and the proposed technique's flow chart.

### Architecture and Working of ANFIS-Based Systems

**Figure 1:**
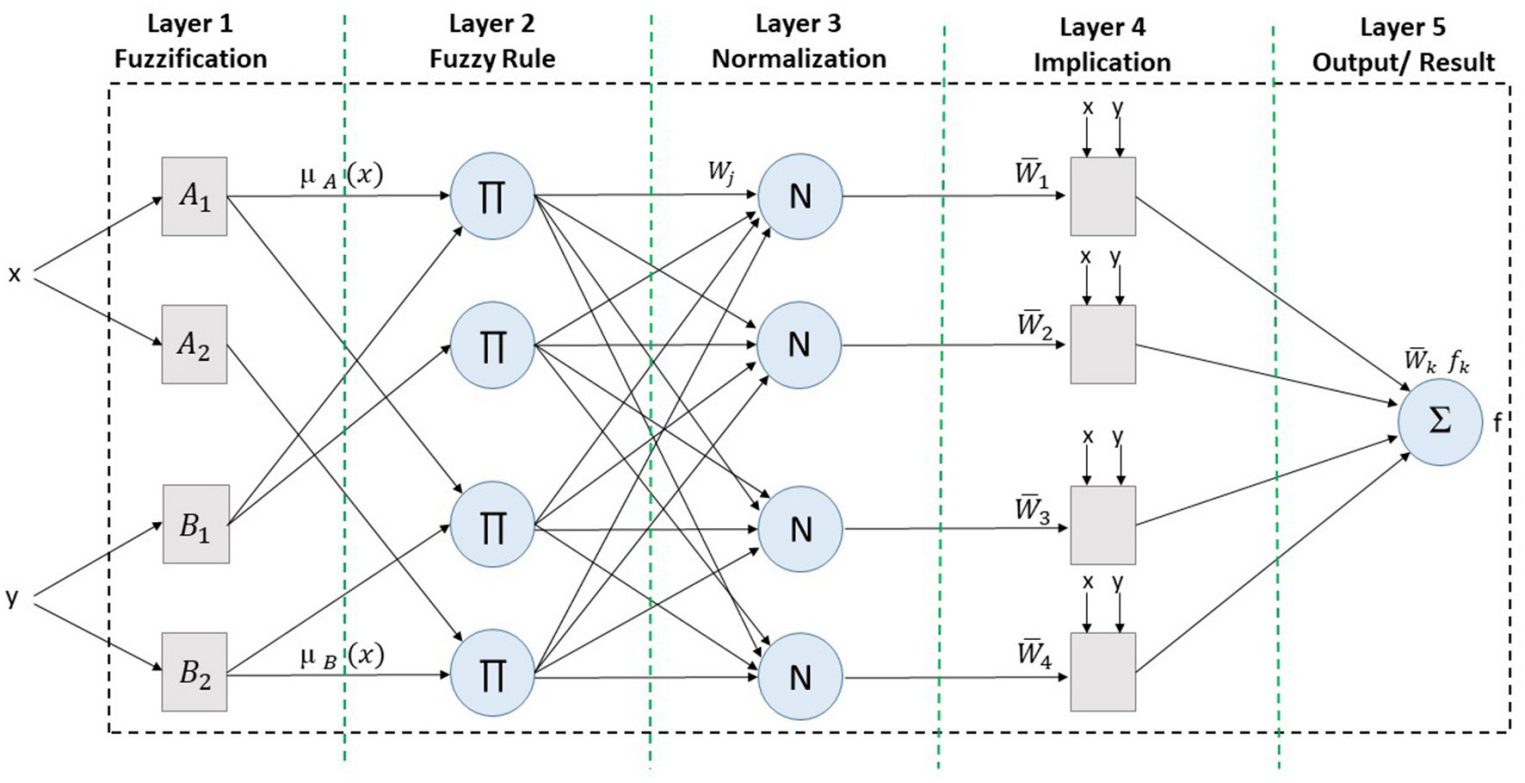
Five layer-based working of the ANFIS architecture.

To study fuzzy rules-based non-linear systems, the fuzzy inference system (FIS) is considered an efficient methodology. Similarly, artificial neural networks (ANNs) work on neurons and the artificial Intelligence technique. So, an artificial neural fuzzy inference system (ANFIS) is the combination of FIS and ANN, which works on the principle of neural networks and follows the efficiency of FIS. ANFIS uses the hybrid Sugeno-type methodology to specify the input parameters. This method trains the membership function based on input parameters to get the trained dataset. ANFIS is also known for its the parameters which can validate the model. ANFIS is based on a multiple layer architecture, where every layer keeps forwarding the input parameters for continuous working. More detail about the architecture and working of the ANFIS architecture is presented in Figure 1.

The architecture of the ANFIS methodology almost resembles that of the Sugeno-type model, as shown in Figure 1. The model-based rule sets are as follow:

- If x is A_1 and y is B_1 then f_1 = p_1 x + q_1 y + r_1
- If x is A_2 and y is B_2 then f_2 = p_2 x + q_2 y + r_2

The working of each layer is explained with mathematical equations as listed below: Layer 1 shows the input parameters and fuzzification, where Ni (N is node) is adaptive to N func (func is function), as represented in Equations 1 and 2.


(1) $$\begin{array}{l} L_{1},i=\mu_{A}i\left(x\right),for\;i=1,2 \end{array}$$

(2) $$\begin{array}{l} L_{1},i=\mu_{B}i-2\left(x\right),for\;i=3,4 \end{array}$$

Layer 2 shows the fuzzy rule generation phase, where every N computes and sets rules by the multiplication process, as represented in Equation 3.


(3) $$\begin{array}{l} L_{2},i=w_{i}=\mu_{A}i\left(x\right).\mu_{B}i\left(y\right),i=1,2 \end{array}$$

Layer 3 shows the normalization phase, where every neuron is normalized based on the effect of the fuzzy rule sets, as represented in Equation 4.


(4) $$\begin{array}{l} L_{3},i=\bar{w}=\frac{w_{i}}{w_{1}+w_{2}},i=1,2 \end{array}$$

Layer 4 shows the implication phase, where every input value is set as an input parameter, as represented in Equation 5.


(5) $$\begin{array}{l} L_{4},i=\bar{w_{i}}f_{i}=\bar{w_{i}}\left(p_{i}x+q_{i}y+r_{i}\right),i=1,2 \end{array}$$

Layer 5 shows the final result or output of the complete process, where the sum of the input values are computed, as represented in Equation 6.


(6) $$\begin{array}{l} L_{5},i=\Sigma \bar{w}f_{i}=\frac{\Sigma w_{i}f_{i}}{\Sigma w_{i}} \end{array}$$

**Figure 2:**
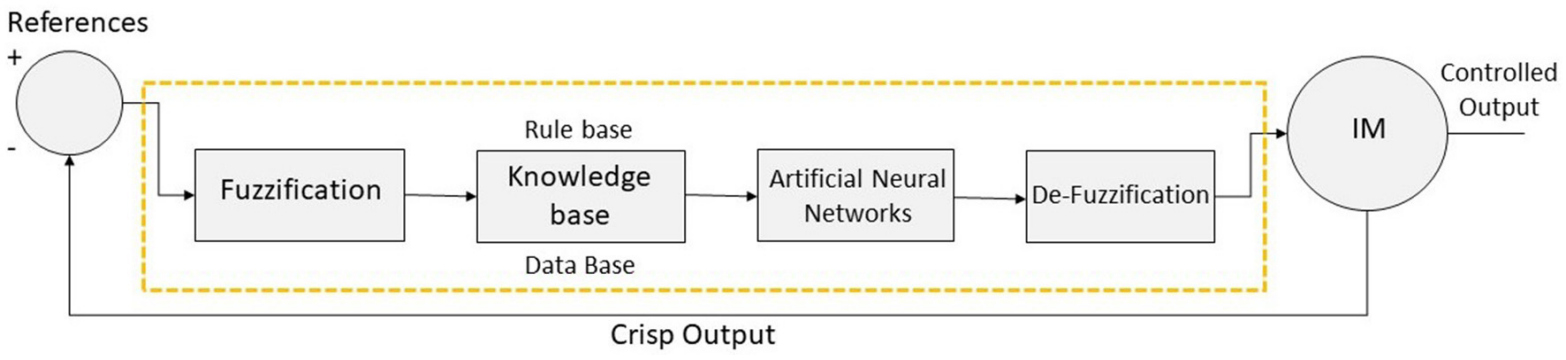
The controller architecture of the ANFIS technique (ANFIS-C).

Figure 2 shows the ANFIS-based controller, which helps the systems detect attacks (ANFIS-C). The controller helps to detect the type of attack based on input parameters and membership function. Through the learning process, ANFIS-C adjusts the initial and processed parameters. The least-square method is used here to obtain hybrid propagation. The ANFIS-C alters the initial and processed parameters based on the error rate (here, error rate is calculated with a difference of two output values before processing and after ANFIS-C).

**Figure 3:**
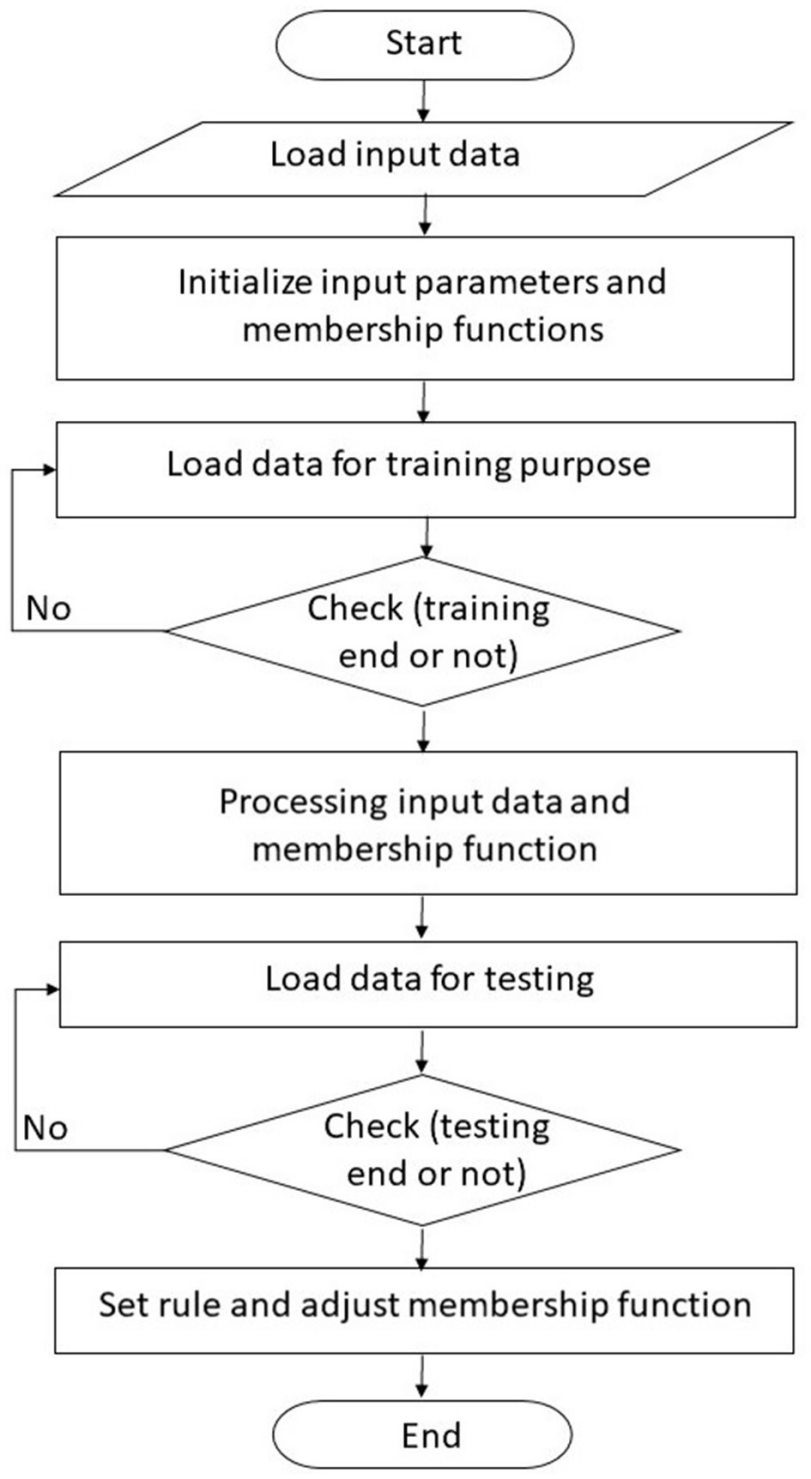
Flow chart of ANFIS-based system.

Figure 3 shows the working of the ANFIS-based intelligence system. The initial step involves loading input parameters, and the ANFIS-based classifier evaluates the functions and detects the error rate, intelligence method, and other learning attributes. After that, the training phase defines the initial and processed membership function along with its parameters. Then, after passing from the testing phase, it sets rules and defines membership functions. At last, the ANFIS-based system stores processed parameters and processed attributes.

## Experimental Analysis and Results

The ANFIS model is used to detect the type of attack based on rule viewer, membership function, and surface viewer. For the practical implementation of the ANFIS model, we use the MATLAB framework for experiments and results. We use dataset pf KDDcup 99 for intrusion detection. This dataset consists of 41 features of the network and five network types of intrusions. The network is observed for around 7 weeks to collate more accurate and precise data. We use an ANFIS-based classifier for detection purposes. The basic structure and features of the ANFIS model in PHR network attacks can be examined. In Table 3 input parameters and membership functions are discussed.

**Table 3:** Input/Output parameters and membership functions.

**Types of attacks**	**Ranges and rules**
[Input 1] [Normal]	Range: [0 1], MFs: 3 (low, medium, high)
[Input 2] [Probe]	Range: [0 1], MFs: 3 (low, medium, high)
[Input 3] [DoS]	Range: [0 1], MFs: 3 (low, medium, high)
[Input 4] [U2R]	Range: [0 1], MFs: 3 (low, medium, high)
[Input 5] [R2L]	Range: [0 1], MFs: 3 (low, medium, high)
[Output] [Attack_type]	Range: [0 1], MFs: 3 (low, medium, high)

**Figure 4:**
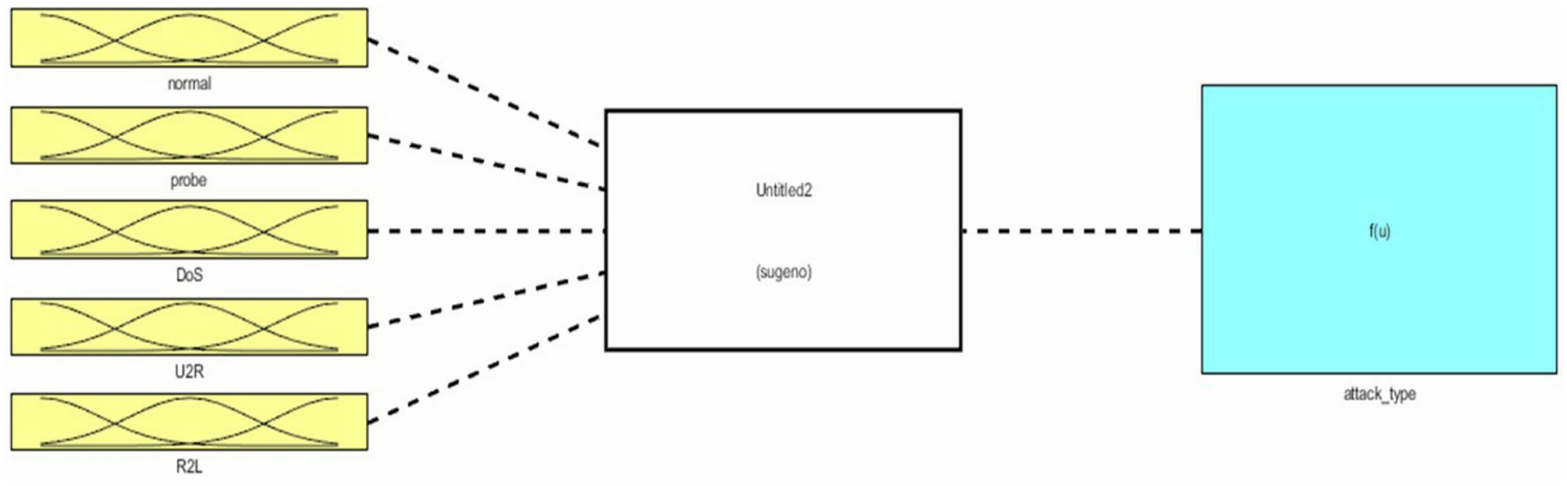
Input parameters.

Similarly, Figure 4 shows the five input parameters (based on attacks type): normal, probe, DoS, U2R, and R2L.

**Figure 5:**
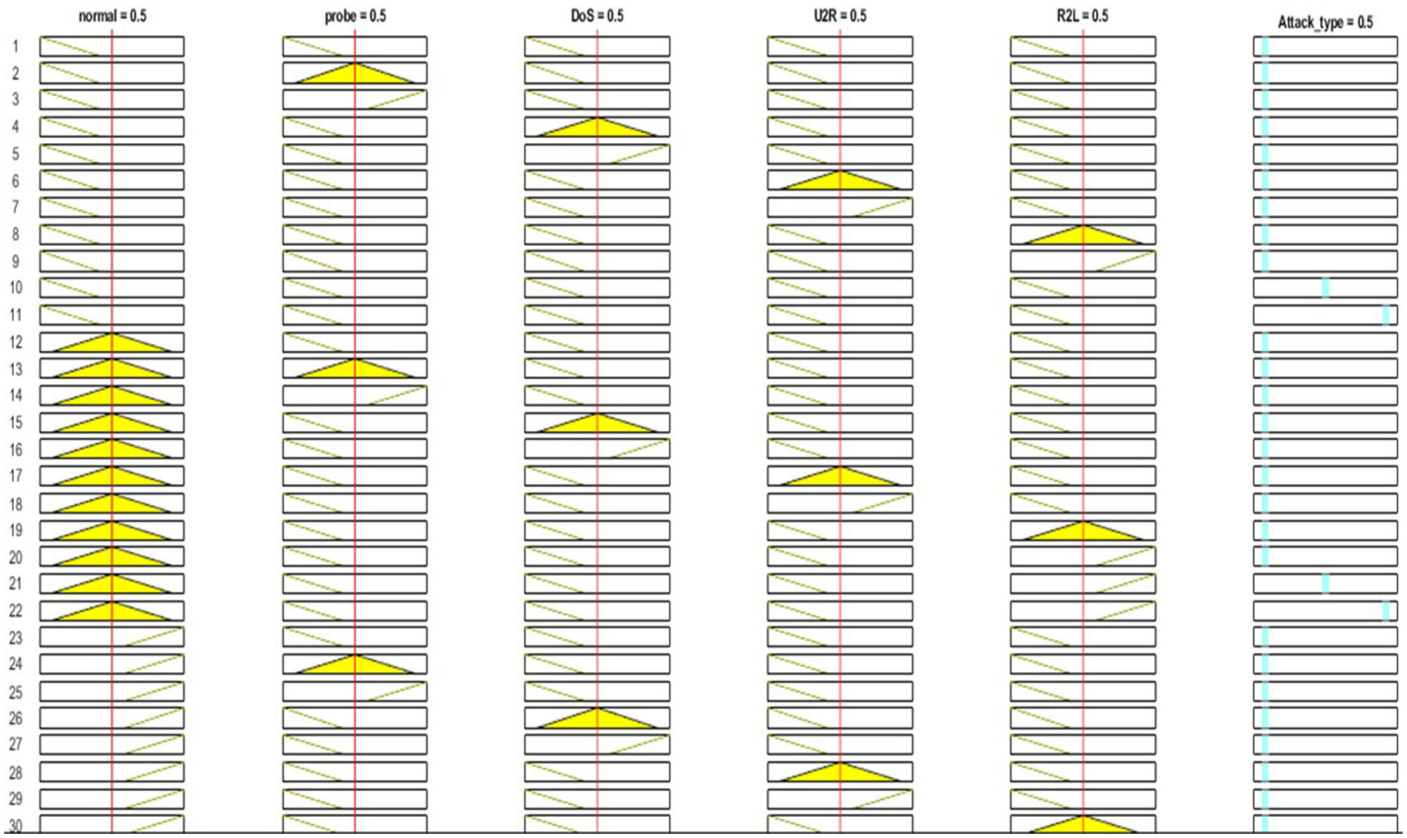
Set of rules based on membership functions.

On the basis of input parameters, membership function is defined using the Sugeno-type ANFIS function in Figure 5. More than 140 rules are generated to generate the desired output value in the form of attack type. After selecting various combinations of input parameters, various rules are generated to get the results.

**Figure 6:**
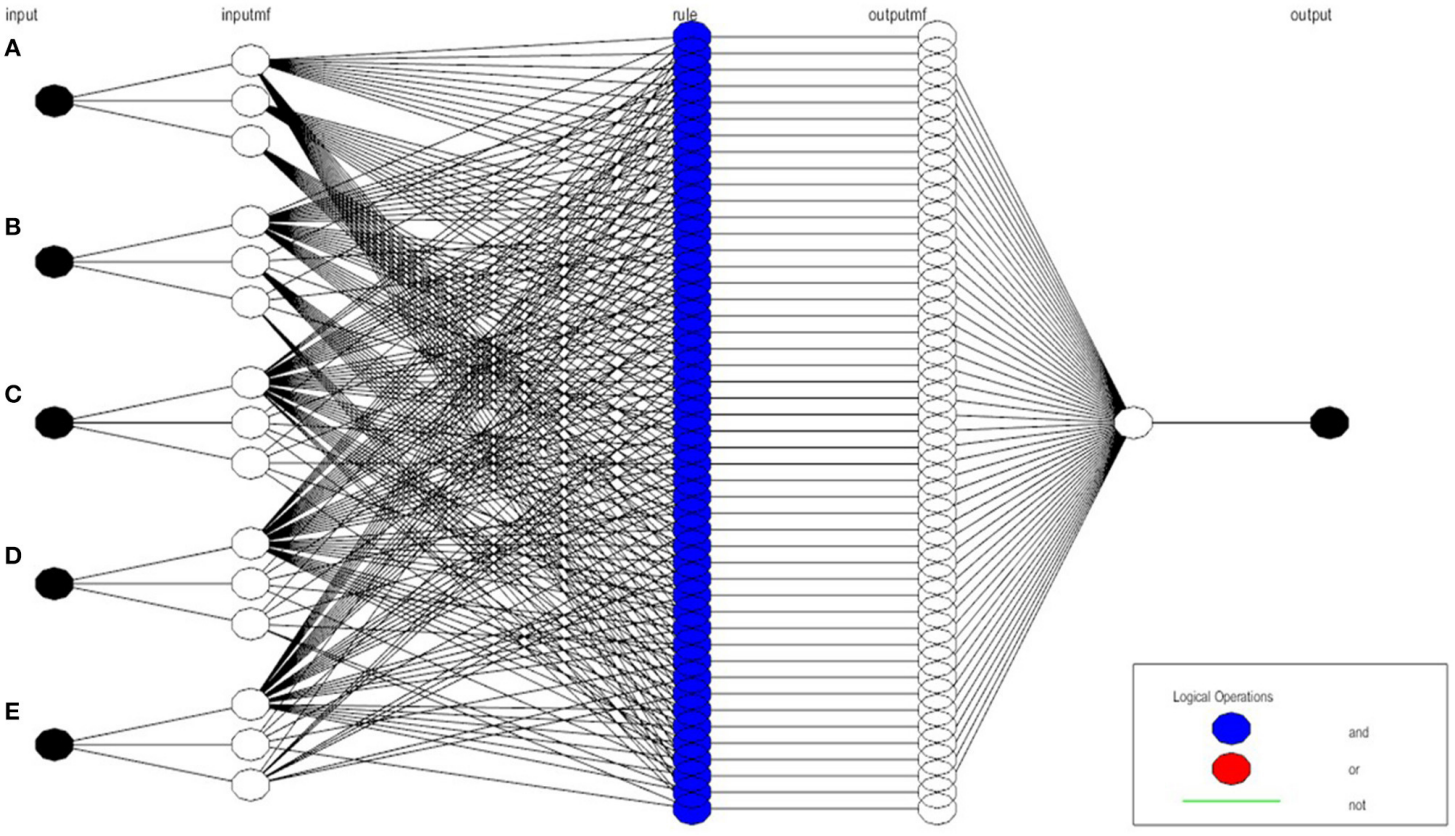
**(A–E)** Set of rules based on membership functions.

Figure 6 shows the 3D surface views of all attributes. In section (Figure 6A), for attack type the x-axis displays “normal” and the y-axis shows “probe”; then in section (Figure 6B), the x-axis presents “probe” and the y-axis shows “normal”; in section (Figure 6C), the x-axis shows “U2R” and the y-axis shows “normal”; then in section (Figure 6D), the x-axis displays “DoS” and the y-axis presents “normal”; and lastly in section (Figure 6E), the x-axis displays “R2L” and the y-axis shows “normal.” The surface view shows the comparison of multiple attack type attributes. As the KDD99cup dataset consists of many types of network attacks. In our research, we choose five types of attacks based on their repetition. Moreover, the surface viewer helps predict the accuracy rate in classification and detection based on a rule (which is created by using the membership function).

**Figure 7:**
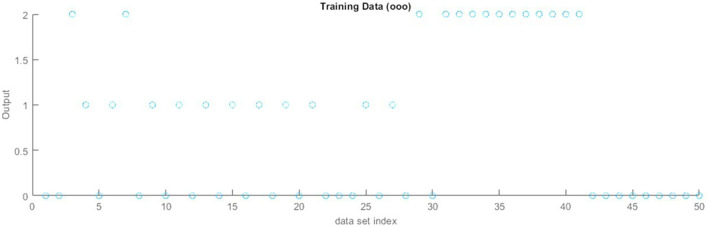
Proposed ANFIS model.

Similarly, after defining rule sets, the structure of the proposed ANFIS-based model is established, as can be seen in Figure 7. In the final structure, after multiple input values, around 140 sets of rules are generated which leads toward the final output value, which indicates the type of intrusion attack.

**Figure 8:**
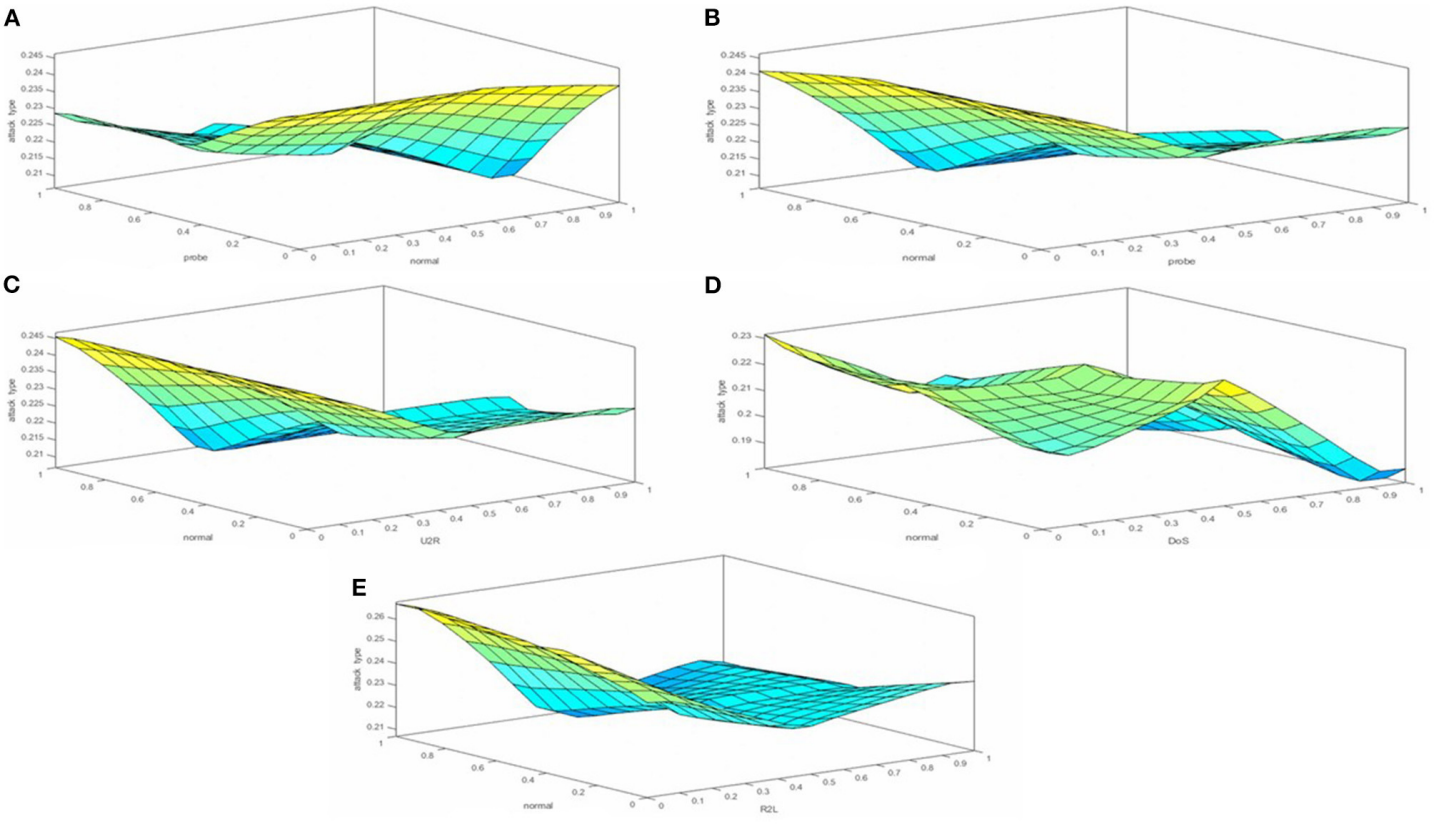
Training results of the KDDcup 99 dataset. **(A)** On x-axis “normal”. **(B)** On x-axis “probe”. **(C)** On x-axis “U2R”. **(D)** On x-axis “DoS”. **(E)** On x-axis “R2L”.

Figure 8 represents the training results of the KDDcup 99 datasets collected from the Kaggle website for network intrusion detection based on ANFIS. The training model contains five input values and generates a single output. The training model shows the increasing flow of accuracy rate.

Finally, Table 4 shows the detailed statistical result of the KDDcup 99 datasets. The selected classes for testing and training are normal, probe, DoS, R2L, and U2R.

**Table 4:** Testing and training results from the KDDcup 99 datasets.

**Types of attacks**	**Details**	**Training result**	**Testing result**
Normal	Stable connectivity	97,278	60,593
Probe	Configuration and analysis details of the system and network	4,107	4,166
DoS	Affecting network resources	391,458	229,853
U2R	Accessibility to servers and connected nodes	52	258
R2L	Illegal accessibility to remote devices	1,126	16,189

The final result generated by the ANFIS model is either linear or non-linear. Therefore, the Sugeno-type ANFIS model helps to obtain a single output. The results show that the PHR in the healthcare system require an ANFIS-type framework to detect and prevent network attacks and maintain secrecy and a trustful environment for patients, doctors, laboratories, and all other interlinked sectors.

### Comparison of Experimented FIS and ANFIS Model Decision-Making

Table 5 shows the performance comparison of the FIS and ANFIS models for better decision-making in network intrusion detection to make e-healthcare (PHR) more secure. The FIS model has been selected to conduct another experiment to show the comparison of the results of FIS and ANFIS based on performance. The mentioned table helps to evaluate the performance of both models. Other than selecting already proposed solutions, we train the FIS model on the same datasets to obtain results. The computed MSE value for FIS is 0.0183 and 0.0123 for ANFIS, the NMSE value is 0.3185 for FIS and 0.2650 for ANFIS, the MAE value is 0.1170 for FIS and 0.0747 for ANFIS, the error value (for minimum observation) is 0.0110 for FIS and 0.0021 for ANFIS, the error value (for maximum observation) is 0.1279 for FIS and 0.1706 for ANFIS, and then finally the R-value is 0.6133 for FIS and 0.7336 for ANFIS. Based on the performance results and output values, ANFIS is considered a more efficient model for efficient decision-making in e-healthcare systems.

**Table 5:** Comparison of FIS and ANFIS models.

**Performance**	**FIS**	**ANFIS (Proposed)**
MSE	0.0183	0.0123
NMSE	0.3185	0.2650
MAE	0.1170	0.0747
Error (min. obs)	0.0110	0.0021
Error (max. obs)	0.1279	0.1706
R-value	0.6133	0.7336

## Conclusion

Security of network systems in all sectors has become one of the most crucial challenges for researchers. In smart e-healthcare systems, cloud servers provide a better intermediary platform for data storage, communication, and many other aspects. The end users (doctors, clinical experts, patients, laboratories) get the opportunity to access PHR and access the networking database servers. It can be any authorized person. It is essential to detect and classify malicious activities and network traffic. This research paper proposed ANFIS for effective intrusion detection in healthcare networks to maintain a patient record. The main agenda is to detect the unauthorized access of users by using an ANFIS-based intrusion detection system. The proposed security algorithm helps to collect malicious activities or information from network traffic. Based on the if-then rule statement and ANFIS-based data classification that helps to detect the intrusion attack, it can determine whether the networking database server was hit by an intruder or not. If the system identifies the type of attack, this can help researchers put precautionary measures into place to overcome the loss or block the malicious incoming traffic.

## Data Availability Statement

The original contributions presented in the study are included in the article/supplementary material, further inquiries can be directed to the corresponding author/s.

## Author Contributions

FA and DL: conceptualization. DL: data curation. FA: formal analysis, investigation, and methodology. NK: funding acquisition. NK and PZ: project administration. SA and PZ: resources. FA and MR: software. FA and NK: supervision. DL and SA: validation. DL and NK: visualization. PZ and SA: writing–review and editing. All authors contributed to the article and approved the submitted version.

## Conflict of Interest

The authors declare that the research was conducted in the absence of any commercial or financial relationships that could be construed as a potential conflict of interest. The handling editor declared a past co-authorship with one of the authors MR.

## Publisher's Note

All claims expressed in this article are solely those of the authors and do not necessarily represent those of their affiliated organizations, or those of the publisher, the editors and the reviewers. Any product that may be evaluated in this article, or claim that may be made by its manufacturer, is not guaranteed or endorsed by the publisher.
